# Identification of a critical determinant that enables efficient fatty acid synthesis in oleaginous fungi

**DOI:** 10.1038/srep11247

**Published:** 2015-06-10

**Authors:** Haiqin Chen, Guangfei Hao, Lei Wang, Hongchao Wang, Zhennan Gu, Liming Liu, Hao Zhang, Wei Chen, Yong Q. Chen

**Affiliations:** 1 State Key Laboratory of Food Science and Technology, School of Food Science and Technology, Jiangnan University, Wuxi 214122, P.R. China; 2 The Synergistic Innovation Center for Food Safety and Nutrition, Wuxi 214122, P.R. China; 3 TEDA School of Biological Sciences and Biotechnology, Nankai University, Tianjin Economic-Technological Development Area, Tianjin 300457, P. R. China; 4 Department of Cancer Biology, Wake Forest University School of Medicine, Winston-Salem, NC 27157, USA

## Abstract

Microorganisms are valuable resources for lipid production. What makes one microbe but not the other able to efficiently synthesize and accumulate lipids is poorly understood. In the present study, global gene expression prior to and after the onset of lipogenesis was determined by transcriptomics using the oleaginous fungus *Mortierella alpina* as a model system. A core of 23 lipogenesis associated genes was identified and their expression patterns shared a high similarity among oleaginous microbes *Chlamydomonas reinhardtii*, *Mucor circinelloides* and *Rhizopus oryzae* but was dissimilar to the non-oleaginous *Aspergillus nidulans*. Unexpectedly, Glucose-6-phosphate dehydrogenase (G6PD) and 6-phosphogluconate dehydrogenase (PGD) in the pentose phosphate pathway (PPP) were found to be the NADPH producers responding to lipogenesis in the oleaginous microbes. Their role in lipogenesis was confirmed by a knockdown experiment. Our results demonstrate, for the first time, that the PPP plays a significant role during fungal lipogenesis. Up-regulation of NADPH production by the PPP, especially G6PD, may be one of the critical determinants that enables efficiently fatty acid synthesis in oleaginous microbes.

Oleaginous microbes are of great interest to the energy and food industries. Understanding the mechanisms by which highly efficient lipogenesis is achieved may have a significant impact for the future use of single-cell oils as biodiesels and dietary fat. Oleaginous fungi such as *Mortierella alpina* and *Mucor circinelloides* can accumulate lipids to a level as high as 50% of their cell mass when under nitrogen starvation^1^,^2^. Previously, we have characterized the genome and major lipid products of *M. alpina*^3^. However, genomic data alone could not adequately explain the mechanisms by which *M. alpina* achieves its effective lipid synthesis.

The accumulation of lipid in oleaginous microbes could be considered as a dynamic equilibrium of fatty acid synthesis and degradation. Modifications of some genes in the carbon flux have minor effects on the level of fatty acid synthesis. For instance, gene inactivation or overexpression of fatty acid desaturase altered fatty acid compositions but not the total amounts of lipids^4^,^5^. Overexpression of acetyl-CoA carboxylase in *Aspergillus oryzae* did not significantly increase the total fatty acid level^6^. Overexpression of glycerol-3-phosphate dehydrogenase in *Yarrowia lipolytica* increased fatty acid synthesis, while also enhancing oxidation^7^. NADPH provides the reducing power necessary for fatty acid synthesis. Traditionally, the cytosolic malic enzyme (ME, EC 1.1.1.40) was considered to be the sole provider of NADPH for fatty acid synthesis in oleaginous microbes^8^,^9^,^10^. However, this unique role of ME in microbial lipid synthesis was challenged^11^,^12^ and this enzyme is now recognized as one of the sources of NADPH for fatty acid synthesis^13^,^14^. However, the role of alternative NADPH sources, such as the pentose phosphate pathway^15^ and other NADPH-generating metabolic reactions^14^,^16^, in fatty acid synthesis is not understood. In addition, evidence suggests that β-oxidation may also be important in some microbial lipid accumulation processes^17^.

Due to the complexity of fatty acid metabolism, the analysis of a single gene or pathway is not sufficient to gage the genome-wide, dynamic metabolic process. In the present study, we compared the transcriptomes prior to and after the onset of lipogenesis and examined the coordinated regulation of genes involved in carbon flux and NADPH production using *M. alpina* as a model system, in order to understand better the mechanisms underlying the efficient lipid synthesis and accumulation in oleaginous microbes.

## Results

### Analysis of global expression pattern by transcriptomics

**Figure 1:**
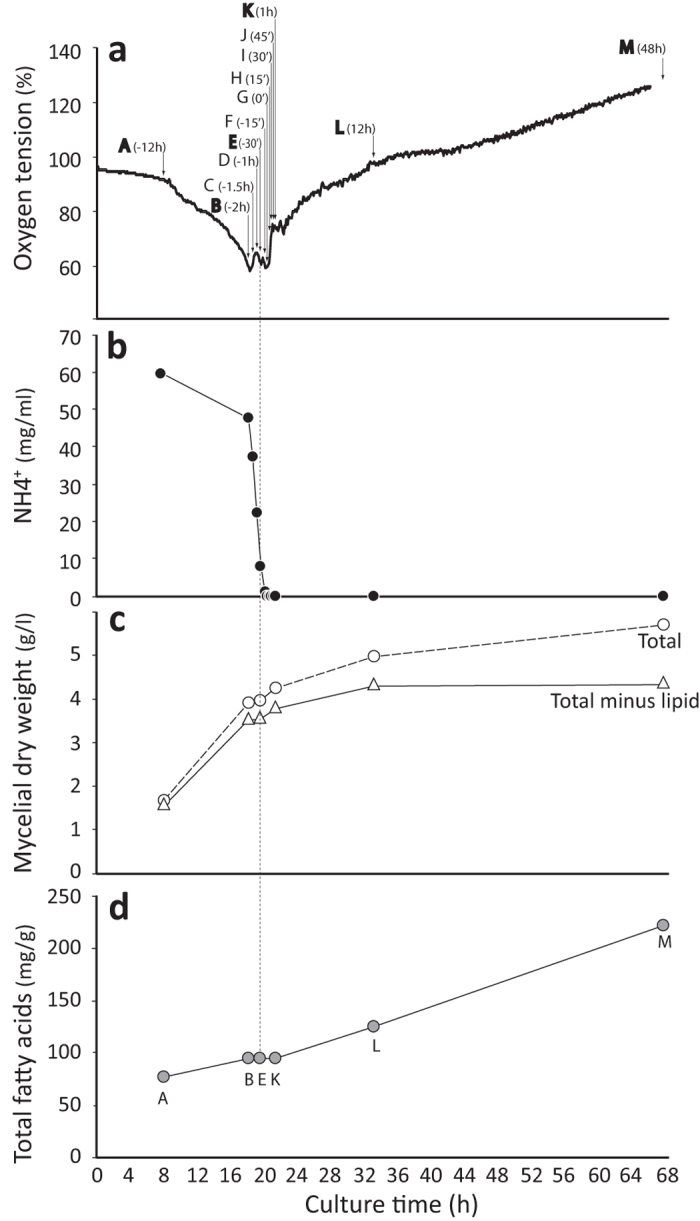
*M*. *alpina* proliferation and lipogenesis. *M. alpina* was cultured in a 3‐L fermenter. Oxygen tension (**a**) was monitored every 6 min. Ammonium concentration in culture medium (**b**) was measured at different time points (A to M). Culture dry weight (**c**) and total fatty acid content (**d**) were determined at various time points (A, B, C, K, L and M). Three independent experiments were performed and the results from one representative experiment are shown. Mycelial weight is shown both as total dry weight and as dry weight with lipid weight subtracted, to illustrate the relative contribution of fatty acids to weight in late time points.

*M. alpina* was cultured in a 3‐L fermenter. As typical, growth of *M. alpina* presented two distinctive phases. Initially fungal mass increased rapidly (Fig. 1) as all nutrients were in excess, reflected by high oxygen tensions (Fig. 1) and ammonium concentrations (Fig. 1). After nitrogen exhaustion, protein and nucleic acid synthesis were severely limited, and thus cell multiplication stopped (Fig. 1); however, glucose continued to be assimilated and the cells now switched to lipid accumulation (Fig. 1). The increase in biomass after this switch was largely due to the accumulation of fatty acids (Fig. 1).

To assess the coordinated transcriptional regulation of lipogenesis, we carried out transcriptome analyses at various time points prior to and after nitrogen exhaustion (sample A: −12 h, B: −2 h, E: −30 min, K: +1 h, L: +12 h and M: +48 h, Fig. 1). Out of all the 12,796 predicted genes, ~88% were expressed; 33% of these expressed genes encode proteins with as yet unknown functions (Table S1). *M. alpina* genes had a very dynamic expression, ranging from undetectable to 73,528 Fragments Per Kilobase of exon per Million fragments mapped (FPKM). Gene transcription changes were compared among the six samples above using sample E (30 min prior to nitrogen exhaustion) as the reference point. Fold change relative to sample E also showed a dynamic range, from 5 to 10-fold (32.8% of differentially expressed genes), 10 to 100-fold (51.6%), 100 to 1,000-fold (13.6%) and greater than 1,000-fold (2%) (Figure S1). Gene expression patterns were closest between sample 2 h (B) and 30 min (E), followed by 12 h (A) prior to nitrogen depletion. Patterns were similarly close between sample 1 h (K) and 12 h (L), followed by 48 h (M) after nitrogen exhaustion. Expression was most dissimilar between proliferative and lipogenic phases, i.e. between samples A/B/E and samples K/L/M. (Figure S1).

### Coordinated regulation of genes involved in carbon flux

**Figure 2:**
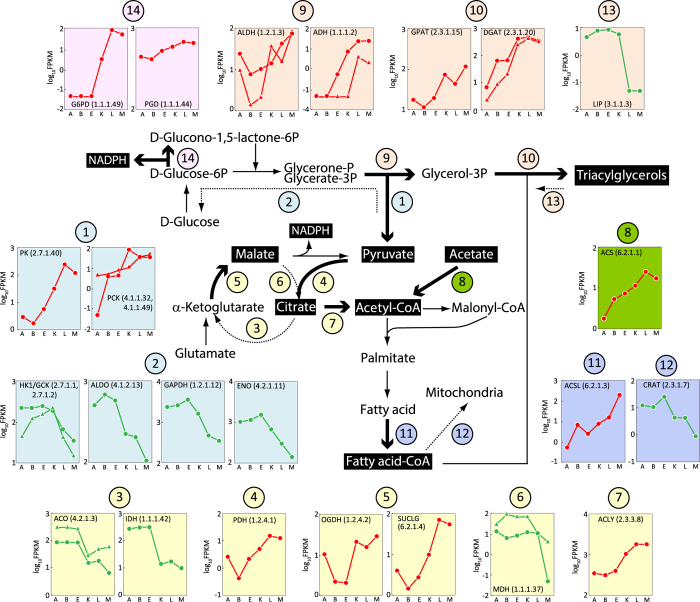
Regulation of de novo lipid synthesis in *M*. *alpina*. Pathways of *de novo* lipid synthesis are illustrated. Major intermediates and final products are highlighted in black, and enzymes found to be regulated at the RNA level during lipid synthesis are indicated by circled numbers. Enzymes involved in glycolysis are highlighted in light blue, pentose phosphate pathway in pink, tricarboxylic acid cycle in yellow, fatty acid synthesis in dark blue and glycerolipid synthesis in orange. Transcriptome analysis was performed in samples from 12, 2 and 0.5 h prior to and 1, 12 and 48 h after nitrogen depletion. RNA expression levels (FPKM in log_10_ scale) for up-regulated enzymes are plotted in red and down-regulated enzymes in green. PK (EC 2.7.1.40): Pyruvate kinase, PCK (EC 4.1.1.32 and EC 4.1.1.49): Phosphoenolpyruvate carboxykinase, HK (EC 2.7.1.1 and EC 2.7.1.2): Hexokinase, ALDO (EC 4.1.2.13): Fructose 1,6-bisphosphate aldolase, GAPDH (EC 1.2.1.12): Glyceraldehyde 3-phosphate dehydrogenase, ENO (EC 4.2.1.11): 2-phosphoglycerate dehydratase, ACO (EC 4.2.1.3): Aconitase, IDH (EC 1.1.1.42): Isocitrate dehydrogenase, PDH (EC 1.2.4.1): Pyruvate dehydrogenase, OGDH (EC 1.2.4.2): Oxoglutarate dehydrogenase , SUCLG (EC 6.2.1.4): Succinyl-CoA synthetase, MDH (EC 1.1.1.37): Malate dehydrogenase, ACLY (EC 2.3.3.8): ATP-citric lyase, ACS (EC 6.2.1.1): Acetyl-CoA synthetase, ALDH (EC 1.2.1.3): Aldehyde dehydrogenase, ADH (EC 1.1.1.2): Alcohol dehydrogenase, GPAT (EC 2.3.1.15): 3-glycerophosphate acyltransferase, DGAT (EC 2.3.1.20): Diacylglycerol *O*-acyltransferase, ACSL (EC 6.2.1.3): Acyl-CoA synthetase, CRAT (EC 2.3.1.7): Carnitine O-acetyltransferase, LIP (EC 3.1.1.3): Triacylglycerol lipase, G6PD (EC 1.1.1.49): Glucose-6-phosphate dehydrogenase and PGD (EC 1.1.1.44): Phosphogluconate dehydrogenase.

The glycolytic pathway breaks down glucose into pyruvate. There was an up-regulation of pyruvate kinase (PK, EC 2.7.1.40) and phosphoenolpyruvate carboxykinase (PCK, EC 4.1.1.32/EC 4.1.1.49) (Fig. 2 ①). This would provide pyruvate for the tricarboxylic acid (TCA) cycle. There was a down-regulation of hexokinase (HK1, EC 2.7.1.1), glucokinase (GCK, EC 2.7.1.2), fructose 1, 6-bisphosphate aldolase (ALDO, EC 4.1.2.13), glyceraldehyde 3-phosphate dehydrogenase (GAPDH, EC 1.2.1.12) and phosphopyruvate hydratase (ENO, EC 4.2.1.11), which would reduce gluconeogenesis to prevent the loss of pyruvate (Fig. 2  ②).

Another regulation involves the TCA cycle. Aconitase (ACO, EC 4.2.1.3) and isocitrate dehydrogenase (IDH, EC 1.1.1.42) were significantly down-regulated (Fig. 2 ③) whereas pyruvate dehydrogenase (PDH, EC 1.2.4.1) was up-regulated after the onset of lipogenesis (Fig. 2 ④), which would increase citrate concentration. 2-oxoglutarate dehydrogenase (OGDH, EC 1.2.4.2) and succinyl-CoA synthetase (SUCLG, EC 6.2.1.4) were up-regulated (Fig. 2 ⑤), whereas malate dehydrogenase (MDH, EC 1.1.1.37) was down-regulated (Fig. 2 ⑥), which would allow the formation of malate from glutamate.

Transcripts of ATP-citrate lyase (ACLY, EC 2.3.3.8) (Fig. 2 ⑦) and acetyl-CoA synthetase (ACS, EC6.2.1.1) (Fig. 2 ⑧) were increased, which would supply acetyl-CoA for fatty acid synthesis.

Notably, there were no significant changes at the transcriptional level in the above-mentioned genes encoding enzymes directly involved in fatty acid biosynthesis. The transcript levels were relatively high at all time-points: acetyl-CoA carboxylase (ACC, EC 6.4.1.2) ~150 FPKM, fatty acid synthase (FASN, EC 2.3.1.86) ~150 FPKM, fatty acid delta 5 desaturase (FADS5, EC 1.14.19.-) ~1,000 FPKM, two fatty acid delta 6 desaturase (FADS6, EC 1.14.19.3) ~30 and 800 FPKM, three fatty acid delta 9 desaturase (FADS9, EC 1.14.19.1) ~200, 500 and 600 FPKM, fatty acid delta 12 desaturase (FADS12, EC 1.14.19.6) ~1,000 FPKM, and three fatty acid elongases (ELOVL, EC 2.3.1.-) ~90, 130 and 700 FPKM, with the exception of fatty acid omega-3 desaturase (FADS15, 1.14.19.-) which was expressed at low levels (~5 FPKM). Therefore, the availability of precursors rather than the process of fatty acid synthesis appears to be the key target of transcriptional regulation.

Additionally, two groups of enzymes in the glycerolipid pathway were up-regulated: aldehyde dehydrogenase (ALDH, EC 1.2.1.3) and alcohol dehydrogenase (ADH, EC 1.1.1.2), which would supply glycerol-3P (Fig. 2 ⑨); 3-glycerophosphate acyltransferase (GPAT, EC 2.3.1.15) and diacylglycerol *O*-acyltransferase (DGAT, EC 2.3.1.20), which would allow triacylglycerol accumulation (Fig. 2 ⑩). This process may be further facilitated by up-regulation of acyl-CoA synthetase (ACSL, EC 6.2.1.3), preparing fatty acids for esterification (Fig. 2 ⑪), by down-regulation of carnitine *O*-acetyltransferase (CRAT, EC2.3.1.7), preventing fatty acid transport into mitochondria for β-oxidation (Fig. 2 ⑫) and by down-regulation of triacylglycerol lipase (LIP, EC 3.1.1.3) (Fig. 2 ⑬), minimizing triacylglycerol hydrolysis. Expression patterns of the above-mentioned genes were independently confirmed by qRT-PCR.

### Up-regulation of NADPH-generating genes in the pentose phosphate pathway

ME is the key enzyme in the malate/pyruvate cycle generating NADPH from malate^8^. Two ME genes were identified in *M. alpina:* one is identical to the gene coding for isoforms III/IV, which is presumed to be cytosolic and supposed to provide NADPH^9^,^18^ and the other is homologous to the ME gene coding for isoform II in *Mucor circinelloides*, which was considered not to be associated with fatty acid biosynthesis but involved in anaerobic metabolism^19^. The expression level of the gene coding for ME isoforms III/IV was moderate (~50 FPKM) with no significant changes. Glucose-6-phosphate dehydrogenase (G6PD, EC 1.1.1.49) and 6-phosphogluconate dehydrogenase (PGD, EC 1.1.1.44) are enzymes in the pentose phosphate pathway producing NADPH. Transcript for one of the three G6PD homologs identified increased from 0 to 113 FPKM after the onset of lipogenesis (Fig. 2⑭). The expression level of PGD was abundant (~800 FPKM) and increased during lipogenesis (Fig. 2⑭).

### Differences in expression of key genes between oleaginous and non-oleaginous microbes under nitrogen starvation

To evaluate the differences in gene expression between oleaginous and non-oleaginous microbes under nitrogen starvation, the published transcriptome data of microalga *Chlamydomonas reinhardtii*^20^,^21^ and fungus *Aspergillus nidulans*^22^ were re-analyzed. Comparing the expression pattern of the 23 key genes identified in *M. alpina* (Fig. 2), *M. alpina* shared 75% similarity with the oleaginous microalga *C. reinhardtii* and only 36% with the non-oleaginous *A. nidulans* (Figure S2). Interestingly, the expression of G6PD and PGD was up-regulated in the oleaginous microbes *M. alpina* and *C. reinhardtii*, but unchanged in the non-oleaginous *A. nidulans*. This suggests that the pentose phosphate pathway may play a key role during lipogenesis.

### Validation of gene expression patterns in other oleaginous fungi

Since *C. reinhardtii* is a microalga, it is likely that the gene expression patterns will be even more similar among oleaginous fungi. Expression of the 23 key genes was examined in *M. circinelloides* and *Rhizopus oryzae* during lipogenesis by qRT-PCR. Their expression patterns were identical except for MDH (Figure S2 and S3), suggesting a high degree of conservation in the regulation of core genes among these oleaginous fungi.

**Figure 3:**
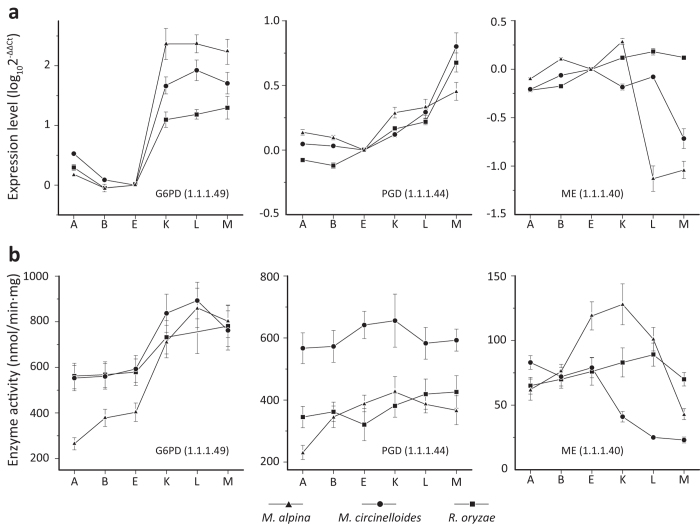
Similarity of coordination of G6PD, PGD and ME expression in oleaginous fungi. Expression level (**a**) and enzymatic activity (**b**) of G6PD, PGD and ME in *M. alpina* (triangles), *M. circinelloides* (circles) and *R. oryzae* (squares). Three independent experiments were performed, and the error bars represent standard deviations.

As in *M. alpina*, G6PD and PGD transcripts were significantly up-regulated in *M. circinelloides* and *R. oryzae*, whereas ME transcripts were decreased during lipogenesis (Fig. 3a). The specific enzymatic activities of G6PD, PGD and ME paralleled their transcriptional expression (Fig. 3b).

### Simulation of NADPH metabolism during *M. alpina* lipogenesis

To gain further insights on NADPH metabolism, a genome-scale metabolic model (GSMM) named iCY1106 was used to analyze overall NADPH anabolic and catabolic reactions during *M. alpina* lipogenesis. According to flux balance analysis (FBA) results at minimal proliferation rate (μ = 0.03), G6PD and PGD were the most significant NADPH producers among the 35 NADPH anabolic reactions identified (Table S2). Fatty acid synthesis reactions were the major NADPH consumers among 135 catabolic reactions identified (Table S3). Minimization of metabolic adjustment (MOMA) analyses suggested that deletion of G6PD and PGD would cause the production of arachidonic acid (AA), the most abundant fatty acid in *M. alpina*, to fall to zero, whereas deletion of 1-pyrroline-5-carboxylate dehydrogenase (EC 1.5.1.12, ALDH4A1), nitrite reductase (EC 1.7.1.4, NIT-6) or hydroxymethylglutaryl-CoA reductase (EC 1.1.1.34, HMGCR) would have no effect, and deletion of pyrroline-5-carboxylate reductase (PYCR) and N-Acetyl-gamma-glutamyl-phosphate reductase (argC) would lead to 32.60% and 45.43% reductions in AA synthesis, respectively (Figure S4).

### Effects of G6PD and PGD knockdown on fatty acid synthesis

**Figure 4:**
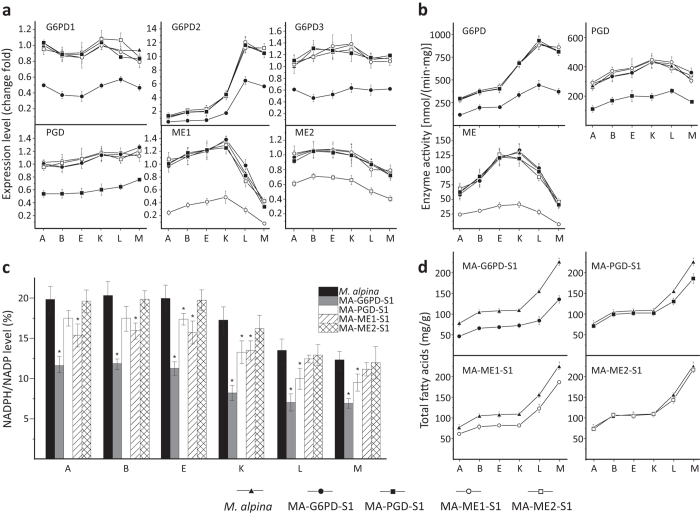
Effect of RNAi targeting G6PD, PGD and ME, in *M*. *alpina*. The expression level (**a**), enzymatic activity (**b**), NADPH level (**c**) and total fatty acid level (**d**) in *M. alpina* strains after RNAi targeting ME, G6PD and PGD. *M. alpina* (up-triangles), wild type *M. alpina*. MA-ME1-S1 (open circle), *M. alpina* malic enzyme 1 silenced strain; MA-ME2-S1 (open square), *M. alpina* malic enzyme 2 silenced strain; MA-G6PD-S1 (circles), *M. alpina* glucose-6-phosphate dehydrogenase co-silenced strain; MA-PGD-S1 (squares), *M. alpina* phosphogluconate dehydrogenase silenced strain. Strains were cultured and sampled as performed in the transcriptome experiment above. Three independent experiments were performed, and the error bars represent standard deviations. *p < 0.05 compared to the wild type.

To confirm the role of G6PD and PGD during lipogenesis, G6PD1, G6PD2, G6PD3, PGD, ME1 and ME2 were knocked down in *M. alpina* by RNA interference (RNAi). The expression of three G6PD isoforms was co-silenced using RNAi targeting common coding sequences, whereas ME1 and ME2 were targeted individually (Figure S5). The desired genes were specifically knocked down without affecting the other genes under investigation (Fig. 4a). Expression was suppressed by 50%–70% and specific enzyme activities were also decreased by approximately the same degrees (Fig. 4b). The suppression of G6PD resulted in a decrease in NADPH level of approximately 40% (Fig. 4c), along with a significant decrease in fatty acid content (Fig. 4d). Knockdown of PGD and ME1 had a lesser effect on NADPH level and fatty acid accumulation (Fig. 4c,d). Interestingly, knockdown of ME2 had little effect on NADPH level (Fig. 4c) or fatty acid synthesis (Fig. 4d).

## Discussion

In the present study, we compared the global gene expression patterns prior to and after the onset of lipogenesis in *M. alpina* and analyzed the expression changes in carbon flux and NADPH metabolism. A core of 23 lipogenesis associated genes was identified. A coordinated regulation of genes involved in carbon flux was noticeable. Unexpectedly, we found that the PPP is the NADPH producer responding to lipogenesis in *M. alpina*. It is known that G6PD and PGD within the PPP generate NADPH; however, their role in fungal lipogenesis is not understood. In fact, cytosolic ME was considered as the sole provider of NADPH for fatty acid synthesis in oleaginous microbes^8^,^9^,^10^. Our results, for the first time, demonstrate that the PPP plays a significant role during *M. alpina* lipogenesis.

The onset of *M. alpina* lipogenesis is triggered by nitrogen starvation. Interestingly, the expression levels of G6PD and PGD were up-regulated in the oleaginous microalga *C. reinhardtii*, but unchanged in the non-oleaginous *A. nidulans* under nitrogen starved conditions (Figure S2). Similar up-regulation was also observed in two other oleaginous fungi, *M. circinelloides* and *R. oryzae* (Fig. 3). Therefore, up-regulation of G6PD and PGD is not limited to *M. alpina* and may be a general phenomenon during microbial lipogenesis.

ME was proposed as the sole provider of NADPH for fatty acid synthesis in oleaginous microbes^8^,^9^,^10^, which was later questioned^11^,^12^. ME and G6PD have different expression patterns during *M. alpina* lipogenesis, i.e. ME expression and activity were high prior to lipogenesis and in the early phase, but then decreased in the later phase of lipogenesis, whereas G6PD expression and activity increased in response to lipogenesis (Fig. 3). PGD expression was also increased, albeit to a lesser degree compared to G6PD during lipogenesis. However, G6PD may play a more significant role than PGD and ME in fatty acid synthesis. Knockdown of G6PD, PGD or ME1 all reduced the level of total fatty acid. This reduction was much more pronounced for G6PD despite a lower knockdown efficiency (approx. 50%) compared to ME (approx. 80%) (Fig. 4a,d). This is probably due to a higher NADPH-producing enzymatic activity of G6PD compared to PGD and ME (Fig. 4b,c).

NADPH is required for numerous cellular processes. Many enzymes such as ME, G6PD, PGD and isocitrate dehydrogenase (IDH) can contribute NADPH, however the mechanisms underlying how organellar pools of NADPH are maintained remain poorly understood. NADP-reducing enzyme responses vary under different stresses. For instance, the contributions of G6PD and IDH seem to be accentuated by oxidative stress, whereas the role of ME is enhanced by starvation in *Drosophila melanogaster*^23^. Our data suggest that G6PD is the major contributor to the NADPH pool during fungal lipogenesis under nitrogen starvation. There are three isozymes of G6PD in *M. alpina*, and only G6PD2 responds to lipogenesis. The relative contribution of each G6PD to the NADPH pool is unclear. The three isoforms could be involved in different aspects of fatty acid synthesis depending on their intracellular localization. We have shown previously that overexpression of cytosolic ME1 increases the level of total fatty acid accumulation^13^. Whereas, overexpression of mitochondrial ME2 increases the level of fatty acid unsaturation without affecting fatty acid accumulation. The NADPH generated by ME2 cannot be transported to the cytosol for fatty acid synthesis or the ME2 produced NADPH is consumed immediately by fatty acid desaturases^24^. We believe that fatty acid synthesis will likely increase when G6PD and PGD are constitutively expressed.

Recently, it was reported that inactivation of the *POX1-POX6* and *PEX10* genes significantly improved fatty acid accumulation in *Y. lipolytica*^17^, suggesting that β-oxidation may be important in microbial lipid accumulation. However, our results indicate no significant changes in expression levels of genes involved in beta-oxidation during *M. alpina* lipogenesis, except for carnitine *O*-acetyltransferase, which was down-regulated, presumably to prevent fatty acid transport into mitochondria for β-oxidation (Fig. 2 ⑫). Therefore, fatty acid loss due to β-oxidation is probably not be a significant issue in *M. alpina*.

The current study identifies NADPH producer genes in the PPP, especially G6PD, as transcriptional up-regulation targets after nitrogen exhaustion in oleaginous fungi. However, some questions remain unresolved. How is this transcriptional regulation achieved? Why is G6PD instead of other NADPH-generating enzymes responding to nitrogen starvation? Addressing these issues at the molecular level will not only improve our understanding of the fundamentals in fungal lipogenesis but also facilitate future genetic engineering of oleaginous microorganisms.

## Methods

### Fungal culture

*Mortierella alpina* (ATCC 32222), *M. circinelloides* and *Rhizopus oryzae* were inoculated on PDA plates (BD Difco^TM^ Potato Dextrose Agar, cat# 213400) and incubated for 20‐30 days at 25 °C. Five ml of modified Kendrick medium (per L: 50 g glucose, 2 g diammonium tartrate, 7.0 g KH_2_PO_4_, 2.0 g Na_2_HPO_4_, 1.5 g MgSO_4_.7H_2_0, 1.5 g Bacto yeast extract, 0.1 g CaCl_2_·2H2O, 8 mg FeCl_3_·6H2O, 1 mg ZnSO_4_·7H_2_O, 0.1 mg CuSO_4_·5H_2_O, 0.1 mg Co(NO_3_)_2_·6H_2_O and 0.1 mg MnSO_4_·5H_2_O, pH 6.0) was added to three plates. Spores were gently scraped off the surface with a sterile loop, and then passed through a 40 micron cell strainer. One ml of spore suspension was added into 50 ml of medium in a 250‐ml flask covered with 8 layers of cheese cloth, and shaken at 200 rpm, 25 °C for 4 days. Mycelia were collected, weighed and blended in fresh medium (0.25 g/ml) using a Braun hand blender for 8 pulses of 5 s each. A half ml of mycelial suspension was inoculated into 50 ml of medium in a 250‐ml flask and shaken at 200 rpm, 25 °C for 36 h. The blending procedure was repeated once and one ml of mycelial suspension was inoculated into 100 ml of medium in a 500‐ml flask and shaken at 200 rpm, 25 °C for 36 h. Two hundred ml of the culture were inoculated into 1500 ml of modified Kendrick medium in a 3‐L fermenter (New Brunswick Scientific Co., Inc., Edison, New Jersey) and agitated at 500 rpm at 25 °C. Dissolved oxygen tension was monitored with the built‐in electrode. Ammonium concentration was determined as described previously^25^. Mycelia were collected at different time points. One fifth of each sample was used for transcriptome analysis. The remainder was lyophilized, weighed and used for total fatty acid analysis.

### Transcriptome analysis

Paired RNA-sequencing reads of 100 bp in length were obtained from the Illumina GA IIx sequencing platform, and all of the raw data were deposited in Sequence Read Archive database (http://www.ncbi.nlm.nih.gov/Traces/sra/) under the accession numbers of SRR1638088, SRR1638089, SRR1638091, SRR1638092, SRR1638093, and SRR1638095. Reads were trimmed from the end to 75 bp and mapped to coding sequences extracted from the annotated *M. alpina* genome using Bowtie^26^ version 0.12.7 with no mismatches on end-to-end hits and the maximum insert size for paired-end alignment set as 500 bp. Average and standard deviation of the insert size for each sample were calculated from the alignment file in the SAM format generated by Bowtie. Alignment of paired 75 bp reads to the *M. alpina* genome were performed by TopHat^27^ version 1.1.4 with min-intron-length set as 20, coverage-search, microexon-search, closure-search set as on, mate-inner-dist and mate-std-dev set according to the calculated average and standard deviation of the insert size from the coding sequence alignment. Transcript abundances were calculated using Cufflinks^28^ version 0.9.3 with corresponding genomic regions of annotated genes as input reference annotation and the output normalized expression values in FPKM (Fragments Per Kilobase of exon per Million fragments mapped) analogous to single-read RPKM^29^ were used for further comparative analysis. The smallest non-zero FPKM was 0.0513. Therefore, 0.05 FPKM was considered as the detection limit and used to replace 0 for log_10_ transformation. Sample E was used as the reference point for calculation of fold changes. Hierarchical clustering was performed using the Genesis software as described previously^30^.

### qRT-PCR

Total RNA was isolated from *M. alpina, M. circinelloides* and *Rhizopus oryzae* strains and reverse-transcribed with the PrimeScript RT reagent kit (Takara, Otsu, Shiga, Japan) according to the manufacturer’s instructions. QPCR was performed on an ABI-Prism 7900 sequence detection system (Applied Biosystems, Foster City, CA) with Power SYBR Green PCR Master Mix (Applied Biosystems, Foster City, CA) according to the manufacturer’s instructions. Reaction mixtures were composed of 10 μL of SYBR Green PCR Master Mix, 0.5 μL of each primer pair, 8 μL of distilled water, and 1 μL of DNA template or distilled water as a no-template control. The PCR cycling conditions were 50 ^o^C for 2 min, 95 ^o^C for 10 min, followed by 40 cycles of amplification at 95 ^o^C for 15 s and 60 ^o^C for 30 s. The expression of the internal control gene (18S rRNA) was used as the normalization standard for gene expression.

### Simulation and analysis

The GSMM iCY1106 used for prediction and analysis was reconstructed by our lab according to a published protocol^31^. FBA and MOMA were algorithms integrated in COBRA Toolbox^32^ with Gurobi as solver^33^. FBA^34^ was used for the calculation of flux distribution with the AA exchange reaction as objective function. MOMA^35^ was used to simulation the effect of gene deletion on AA production compared with the wide type strain. All the constraint conditions for simulation were based on an *in silico* medium containing basic elements and 20 regular amino acids^36^.

### RNA interference (RNAi)

Hairpin RNAs were expressed using the binary vector pBIG2-ura5s-ITs^24^. Target gene-specific sequences were amplified from cDNA of *M. alpina*, digested with appropriate restriction enzymes, inserted into the multi-cloning sites located upstream and downstream of the ITs sequence with forward and reverse orientation, respectively. Successful cloning was confirmed by sequencing. Vectors were electro-transformed into *Agrobacterium tumefaciens C58C1*. *A. tumefaciens*-mediated transformation of *M. alpina* was performed as described previously^13^. Spores of *M. alpina* uracil auxotrophic strain CCFM 501 were harvested and centrifuged at 12,000 × g for 20 min and washed once with 10 ml of fresh liquid IM. The pellet was diluted to 10^8^/ml with fresh liquid IM before use. A single-bacteria colony of *A. tumefaciens* C58C1 harboring corresponding binary vector was cultured at 28 °C, shaking at 200 rpm for 48 h in 20 ml of MM liquid medium containing 100 μg kanamycin/ml and 100 μg rifampicin/ml. Cells were harvested by centrifugation at 4,000 × g for 5 min, washed once and diluted to a concentration of 0.3 OD_600_ with fresh IM. The cells were incubated at 28 °C for 8 to 12 h with shaking at 200 rpm to 1.2 OD_600_. The *A. tumefaciens* suspension was mixed 1:1 (100 μl each) with an *M. alpina* spore suspension and spread on cellophane membranes placed on solid IM plates (containing 0.9 g/L glucose). After incubation at 23 °C for 36 to 48 h in the dark, the membranes were transferred onto uracil-free SC plates supplemented with 50 μg cefotaxime/ml and 50 μg spectinomycin/ml and incubated at 28 °C until colonies appeared. Mycelia were transferred onto uracil-free SC agar plates containing 50 μg cefotaxime/ml and 50 μg spectinomycin/ml, followed by three consecutive subcultures to obtain stable transformants. Insertion of RNAi expression cassettes onto *M. alpina* genome was confirmed by PCR with two pairs of specific primers described before^24^. Successful knockdown of target genes was verified by qRT-PCR.

### Enzyme activity assay

Enzyme activity was determined as described previously^37^,^38^,^39^. Mycelia were collected by filtration, then frozen and ground in liquid nitrogen and then resuspended in an extraction buffer containing 20% (w/v) glycerol, 100 mM KH_2_PO_4_/KOH, pH 7.5, 1 mM benzamidine and 1 mM DTT. After centrifuging at 10,000 × g for 10 min at 4 ^o^C, the supernatant was immediately used for enzyme activity measurement by continuous spectrophotometric assays at 340 nm. The unit of enzyme activity was defined as the amount of enzyme required to produce 1 nmol NADPH per min.

### NADPH level analysis

Mycelia from each sample were collected, frozen, lyophilized, and then ground in liquid nitrogen. The NADP and NADPH levels were determined using the NADP/NADPH Quantification Colorimetric Kit (BioVision, California, USA) according to the manufacturer’s instructions.

### Fatty acid analysis

Total fatty acid analyses were performed as described previously^3^,^8^,^40^.

## Additional Information

**How to cite this article**: Chen, H. *et al.* Identification of a critical determinant that enables efficient fatty acid synthesis in oleaginous fungi. *Sci. Rep.* **5**, 11247; doi: 10.1038/srep11247 (2015).

## Supplementary Material

Supplementary Information
