# Supplementary material for: Identification of a critical determinant that enables efficient fatty acid synthesis in oleaginous fungi
Source: Sci Rep. 2015 Jun 10;5:11247. doi: 10.1038/srep11247 (PMC4462047; doi:10.1038/srep11247)
Supplement: Supplementary Information [file srep11247-s1.pdf]

Identification of a critical determinant that enables efficient fatty acid synthesis in oleaginous fungi

Haiqin Chen, Guangfei Hao, Lei Wang, Hongchao Wang, Zhennan Gu, Liming Liu, Hao Zhang, Wei Chen, and Yong Q. Chen

Fold change	5 - 10	10 - 10^2	10^2 - 10^3	10^3 - 10^4	10^4 - 10^5
Genes	1090	1718	452	55	13

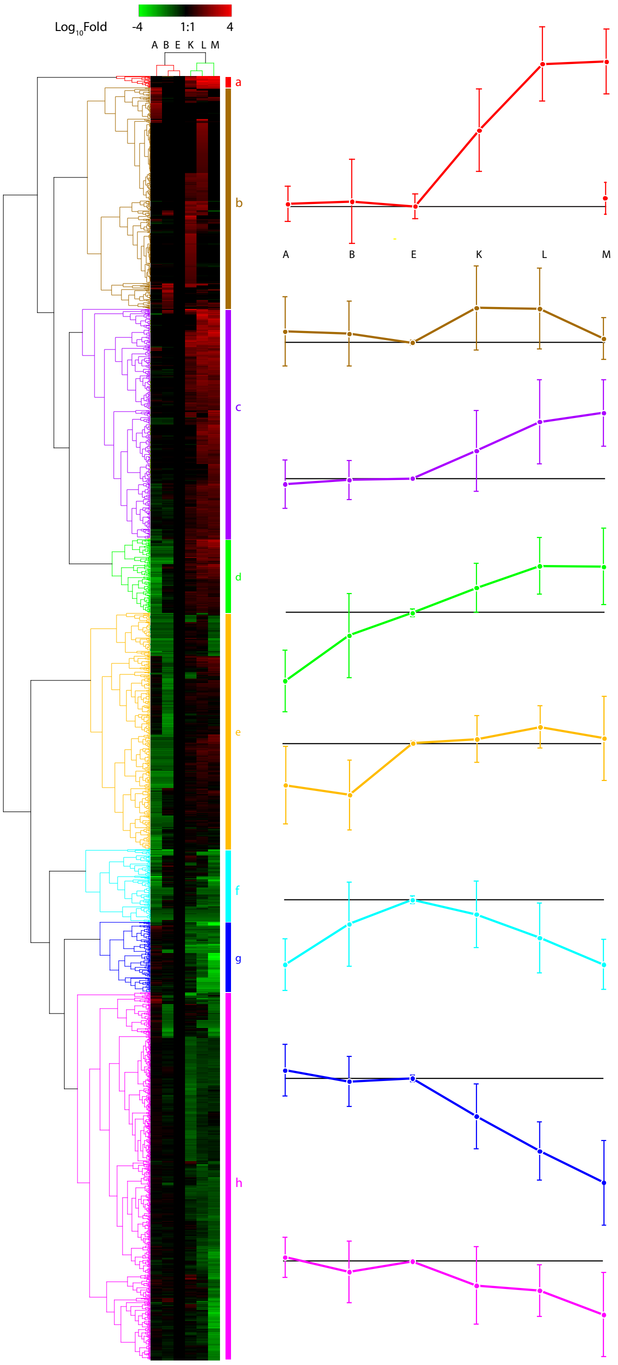

Figure S1 | Overall transcriptional regulation during lipogenesis in *M. alpina*. Gene transcription changes were compared among samples A, B, E, K, L and M using

sample E (30 min prior to nitrogen exhaustion) as the reference point. The smallest non-zero FPKM was 0.0513. Therefore, 0.05 FPKM was considered as the detection limit and used to replace 0 for log10 transformation. The number of genes with >5 fold change is indicated at the top. Hierarchical clustering of differentially expressed genes (3328) and samples (A, B, E, K, L, and M) is shown on the left. Eight gene clusters (a, b, c, d, e, f, g, h) were identified and the average expression pattern for each cluster is shown on the right.

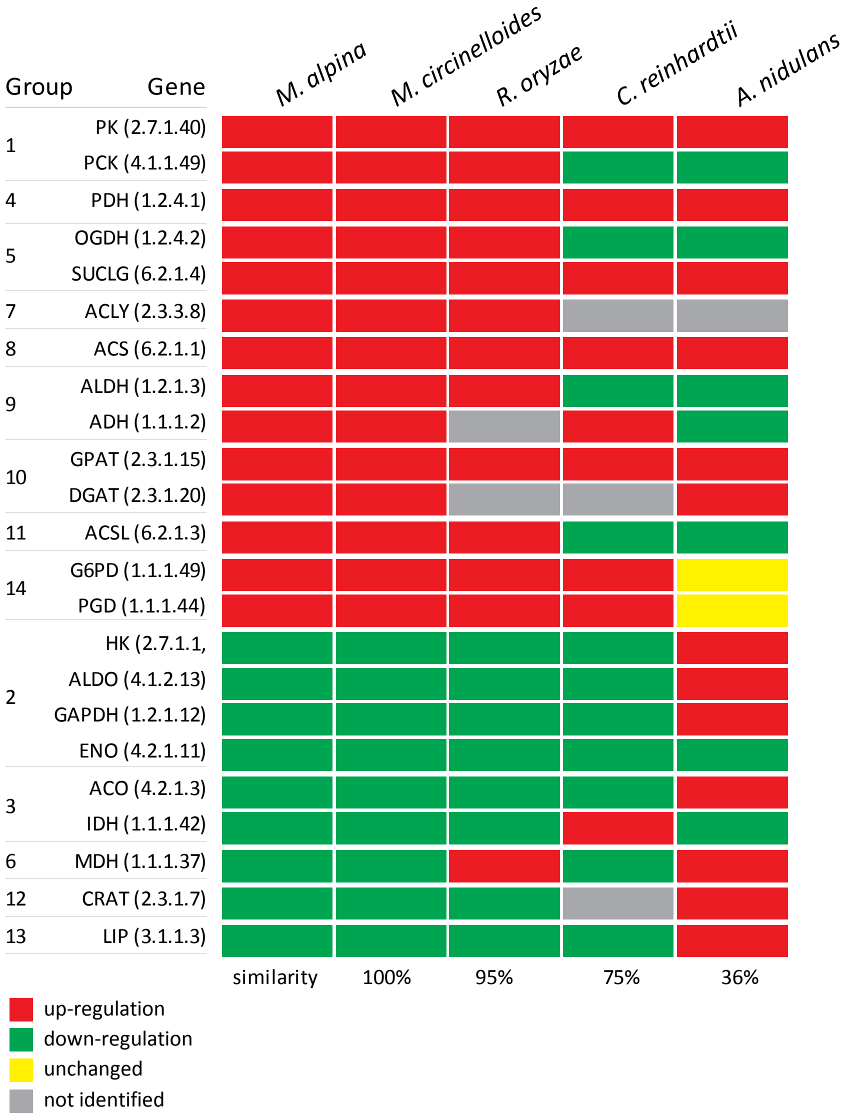

Figure S2 | Similarity in core gene expression. Transcriptional regulation of the 32 core genes involved in carbon flux and NADPH metabolism illustrated in **Figure 2** were compared among *M. circinelloides*, *R.oryzae*, *C. reinhardtii* and *A. nidulans* and *M. alpina*.

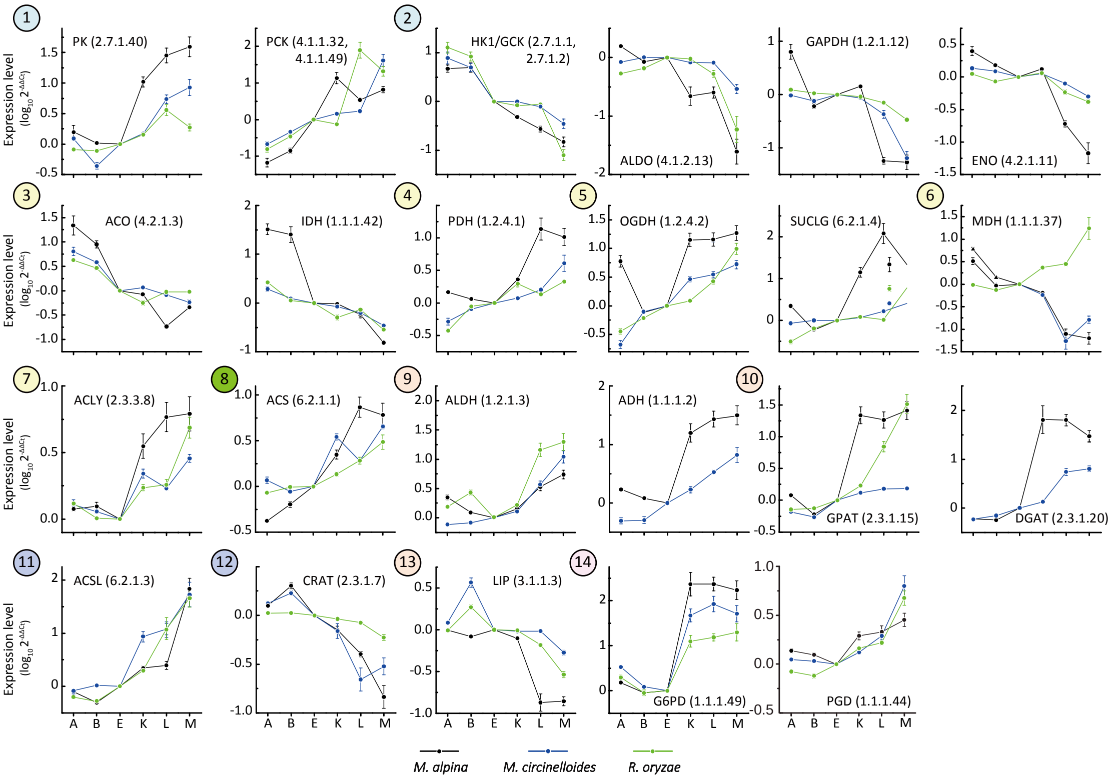

Figure S3 │ Expression similarities of the core genes in *M. alpina*, *M. circinelloides* and *R. oryzae*. The twenty-three genes involved in carbon flux and NADPH metabolism illustrated in **Figure 2** of *M. alpina, M. circinelloides and R. oryzae* were analyzed by qRT-PCR during lipogenesis. Different symbols indicate different genes with the same annotation. The error bars represent standard deviations. The full names of the enzymes are indicated in the legend of **Figure 2**.

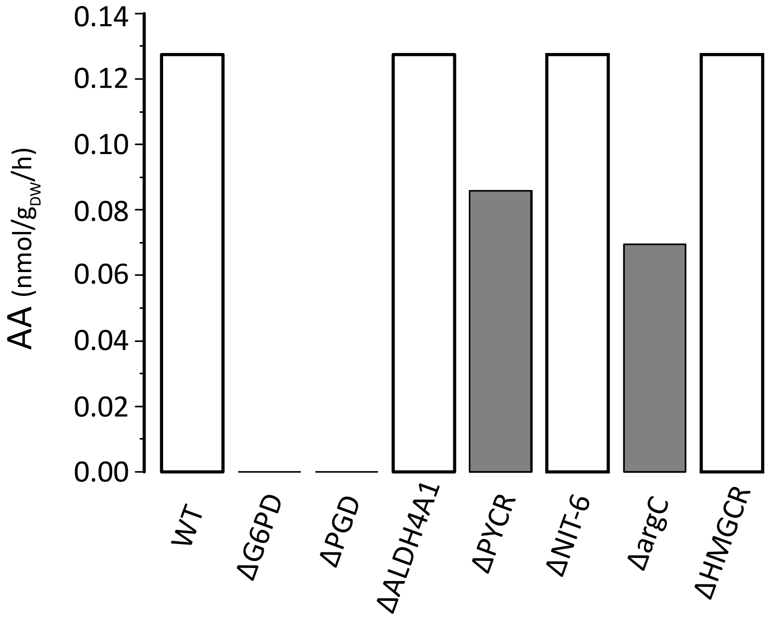

Figure S4 | The effect of different NADPH-generating genes on AA production. The effect of different NADPH generating genes on AA production were simulated by single gene deletion using MOMA. WT: wide type, G6PD: glucose-6-phosphate dehydrogenase (EC 1.1.1.49), PGD: phosphogluconate dehydrogenase (EC 1.1.1.44), ALDH4A1: 1-pyrroline-5-carboxylate dehydrogenase (EC 1.5.1.12), PYCR: pyrroline-5-carboxylate reductase (EC 1.5.1.2), NIT-6: nitrite reductase (EC 1.7.1.4), argC: N-Acetyl-gamma-glutamyl-phosphate reductase (EC 1.2.1.38), HMGCR: hydroxymethylglutaryl-CoA reductase (EC 1.1.1.34).

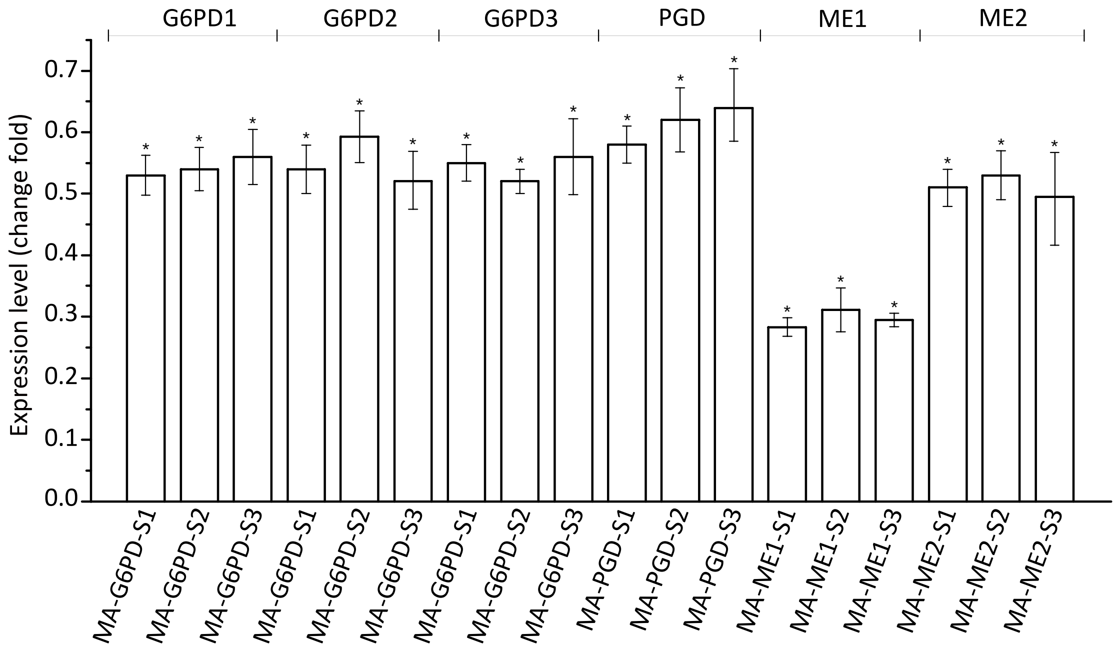

Figure S5 | Confirmation of RNA knockdown. Three mutants from each RNAi experiments were analyzed by qRT-PCR with the wild-type *M. alpina* as control. Samples were taken from cultures growing in 500 mL flasks with 100 mL of Kendrick medium stirred at 200 rpm for 96 h at 28°C. Three independent experiments were performed and the bars represent the standard deviations. *p < 0.05 compared to the wild-type control.

Table S1. Summary of transcriptome data

	Library					
Sample	A*	B	E	K	L	M
Raw RNA sequencing reads (million)	35.73	37.31	56.66	55.60	52.49	33.21
Mapped RNA sequencing reads (million)	27.33	25.24	44.14	45.62	44.47	24.23
Predicted gene models covered	9740	9671	10394	10724	10785	9945

* A: ¬ 12 h, B: -2 h, E: -30 min, K: +1 h, L: +12 h and M: +48 h

Table S2. Predicted NADPH anabolic reactions

Rxn	EC	Enzyme	Formula	μ=0.03
R1	1.2.1.5	aldehyde dehydrogenase [NAD(P)[c] +]	h2o[c] + nadp[c] + acal[c] -> 2 h[c] + nadph[c] + ac[c]	0.004
R2	1.1.1.42	Isocitrate dehydrogenase (NADP[c] +)	nadp[c] + icit[c] -> akg[c] + nadph[c] + co2[c]	0
R3	1.1.1.42	Isocitrate dehydrogenase (NADP[c] +)	nadp[c] + icit[c] -> akg[c] + nadph[c] + co2[c]	0
R4	1.1.1.49	glucose-6-phosphate dehydrogenase	nadp[c] + dg6p[c] <=> h[c] + nadph[c] + d6pgl[c]	1.141
R5	1.1.1.44	Phosphogluconate dehydrogenase (decarboxylating)	nadp[c] + d6pgc[c] -> co2[c] + nadph[c] + rl5p[c]	1.141
R6	1.1.1.40	malate dehydrogenase (oxaloacetate-decarboxylating) (NADP[m] +)	nadp[m] + mal[m] -> pyr[m] + nadph[m] + co2[m]	0.555
R7	1.1.1.40	malate dehydrogenase (oxaloacetate-decarboxylating) (NADP[m] +)	nadp[c] + mal[c] -> pyr[c] + nadph[c] + co2[c]	0
R8	1.1.1.-	unknown	hcoa[m] + nadp[m] -> nadph[m] + h[m] + 3ocoa[m]	0
R9		no ec	malcoa[m] + nadph[m] + 2 h[m] <=> 3ocoa[m] + nadp[m] + h2o[m]	0
R10		no ec	malcoa[c] + nadph[c] + 2 h[c] <=> 3ocoa[c] + nadp[c] + h2o[c]	0
R11	1.1.1.-	unknown	hcoa[m] + nadp[m] -> nadph[m] + h[m] + 3ocoa[m]	0
R12	1.2.1.16	succinate-semialdehyde dehydrogenase	h2o[m] + succsal[m] + nadp[m] -> 2 h[m] + nadph[m] + succ[m]	0
R13	1.1.1.21	L-arabinose reductase	h[c] + nadph[c] + larab[c] <=> nadp[c] + laol[c]	0
R14	1.1.1.21	D-Ribose reductase	h[c] + nadph[c] + rib[c] <=> nadp[c] + ribol[c]	0
R15	1.5.1.12	1-pyrroline-5-carboxylate dehydrogenase	s1p5c[c] + nadp[c] + 2 h2o[c] -> glu[c] + nadph[c] + h[c]	0
R16	1.5.1.12	1-pyrroline-5-carboxylate dehydrogenase	s1p5c[m] + nadp[m] + 2 h2o[m] -> glu[m] + nadph[m] + h[m]	0
R17	1.5.1.12	1-pyrroline-5-carboxylate dehydrogenase	glugsal[c] + nadp[c] + h2o[c] -> glu[c] + nadph[c] + 2 h[c]	0.561
R18	1.2.1.41	glutamate-5-semialdehyde dehydrogenase	2 h[c] + nadph[c] + glup[c] <=> nadp[c] + pi[c] + glugsal[c]	0
R19	1.5.1.12	1-pyrroline-5-carboxylate dehydrogenase	s1p5c[c] + nadp[c] + 2 h2o[c] -> glu[c] + nadph[c] + h[c]	0
R20	1.5.1.2	Pyrroline-5-carboxylate reductase	pro[c] + nadp[c] <=> 2 h[c] + nadph[c] + s1p5c[c]	0.561
R21	1.3.1.13	Prephenate dehydrogenase (NADP[c] +)	nadp[c] + phen[c] -> co2[c] + nadph[c] + 4hppyr[c]	0
R22	1.4.1.21	aspartate dehydrogenase	asp[c] + nadp[c] -> 2 h[c] + nadph[c] + iasp[c]	0.013
R23	1.18.1.2	ferredoxin-NADP+ reductase	nadp[c] + h[c] + refdox[c] -> nadph[c] + oxfdox[c]	0
R24	1.5.1.3	Dihydrofolate reductase	nadp[c] + dhf[c] <=> h[c] + nadph[c] + fot[c]	0
R25	4.2.3.12;1.1.1.153	6-pyruvoyltetrahydropterin synthase/sepiapterin reductase	ahtd[c] + 2 nadph[c] + 2 h[c] -> bh4[c] + 2 nadp[c] + pppi[c]	0
R26	1.5.1.34	6,7-dihydropteridine reductase	bh4[c] + nadp[c] -> bh2[c] + nadph[c] + h[c]	0

R27	1.5.1.5	methylenetetrahydrofolate dehydrogenase (NADP[c] +)	nadp[c] + metthf[c] <=> nadph[c] + methf[c]	0.009
R28	1.3.1.-	Oxidoreductases	ccdol[m] + nadp[m] -> techol[m] + nadph[m] + h[m]	0
R29	1.3.1.-	Oxidoreductases	cdtbe[m] + nad[m] -> dtbe[m] + nadh[m] + h[m]	0
R30	1.1.1.2	alcohol dehydrogenase (NADP[c] +)	6hyac[c] + nadp[c] -> adsde[c] + nadph[c] + h[c]	0
R31	1.2.1.5	aldehyde dehydrogenase [NAD(P)[m] +]	aldmde[m] + nadp[m] + h2o[m] -> capmde[m] + nadph[m] + h[m]	0
R32	1.14.13.1	salicylate hydroxylase	h2o[c] + co2[c] + nadp[c] + ccl[c] <=> o2[c] + 2 h[c] + nadph[c] + sali[c]	0
R33	1.2.1.5	aldehyde dehydrogenase [NAD(P)[c] +]	h2o[c] + nadp[c] + acal[c] -> 2 h[c] + nadph[c] + ac[c]	0.004
R34	1.1.1.170	C-4 sterol decarboxylase (cG26);sterol-4alpha-carboxylate 3-dehydrogenase (decarboxylating)	nadp[c] + dcda[c] -> co2[c] + nadph[c] + cdol[c]	0
R35	1.14.21.6	lathosterol oxidase	epst[c] + nadp[c] -> ergod[c] + nadph[c] + h[c]	0
R36	1.1.1.2;1.1.1.72	NADP-dependent alcohol dehydrogenase/Glycerol dehydrogenase	nadp[c] + gl[c] <=> h[c] + nadph[c] + glyal[c]	0
R37	1.5.1.10	Saccharopine dehydrogenase (NADP[c] +, L-glutamate forming)	h2o[m] + nadp[m] + sacp[m] <=> glu[m] + h[m] + nadph[m] + amasa[m]	0.005

Table S3. Predicted NADPH catabolic reactions

Rxn	EC	Enzyme	Formular	μ=0.03
R1	1.1.1.2	Alcohol dehydrogenase (NADP[c] +)	h[c] + nadph[c] + acal[c] -> nadp[c] + eth[c]	0
R2	1.1.1.271	GDP-L-fucose synthase	gdman[c] + nadph[c] + h[c] -> glfuc[c] + nadp[c]	0
R3	1.1.1.79	glyoxylate reductase (NADP[c] +)	glx[c] + nadph[c] + h[c] -> glya[c] + nadp[c]	0
R4	1.1.1.21	D-Xylose reductase (xyrA) (xylitol dehydrogenase)	h[c] + nadph[c] + xyl[c] -> nadp[c] + xol[c]	0
R5	1.4.1.13	glutamate synthase (NADH)	akg[c] + gln[c] + h[c] + nadph[c] -> 2 glu[c] + nadp[c]	0
R6	1.4.1.3	glutamate dehydrogenase [NAD(P)[m] +]	akg[m] + nh3[m] + nadph[m] + h[m] -> glu[m] + nadp[m] + h2o[m]	0
R7	1.4.1.3	glutamate dehydrogenase [NAD(P)+]	akg[c] + nh3[c] + nadph[c] + h[c] -> glu[c] + nadp[c] + h2o[c]	0
R8	1.1.1.86	(R)-2,3-Dihydroxy-3-methylbutanoate:NADP+ oxidoreductase	hmobut[m] + nadph[m] + h[m] -> nadp[m] + dmbut[m]	0
R9	1.2.1.31	L-aminoadipate-semialdehyde dehydrogenase	2 h[m] + nadph[m] + ama[m] -> h2o[m] + nadp[m] + amasa[m]	0
R10	1.5.1.2	Pyrroline-5-carboxylate reductase	2 h[c] + nadph[c] + phc[c] -> hpro[c] + nadp[c]	0
R11	1.14.13.9	Kynurenine 3-monooxygenase	o2[c] + h[c] + nadph[c] + kyn[c] -> h2o[c] + nadp[c] + hkyn[c]	0
R12	1.1.1.25	shikimate dehydrogenase	h[c] + nadph[c] + dhsk[c] -> nadp[c] + sme[c]	0
R13	1.1.1.86	(R)-2,3-Dihydroxy-3-methylpentanoate:NADP+ oxidoreductase (isomerizing)	rhmopt[m] + nadph[m] + h[m] -> dmvat[m] + nadp[m]	0.006
R14	1.1.1.86	(R)-2,3-Dihydroxy-3-methylpentanoate:NADP+ oxidoreductase (isomerizing)	rhmopt[c] + nadph[c] + h[c] -> dmvat[c] + nadp[c]	0
R15	1.8.1.9	thioredoxin reductase (NADPH)	h[c] + nadph[c] + othio[c] -> nadp[c] + rthio[c]	0
R16	1.8.1.7	glutathione-disulfide reductase	h[c] + nadph[c] + gssg[c] -> nadp[c] + 2 gsh[c]	0
R17	1.7.1.4	nitrite reductase [NADPH]	5 h[c] + 3 nadph[c] + hno2[c] -> h2o[c] + 3 nadp[c] + nh4oh[c]	0
R18	1.8.1.2	Sulfite reductase (NADPH)	3 h[c] + 3 nadph[c] + slfi[c] -> 3 h2o[c] + 3 nadp[c] + h2s[c]	0
R19	1.1.1.65	pyridoxine 4-dehydrogenase	pdxal[c] + nadph[c] + h[c] -> vb6[c] + nadp[c]	0
R20	1.1.1.169	2-dehydropantoate 2-reductase	h[m] + nadph[m] + akp[m] -> nadp[m] + pant[m]	0.003
R21	1.1.1.100	B-ketoacyl-ACP synthase (c10,0), fatty acyl CoA synthase	6 h[c] + 4 nadph[c] + 3 malcoa[c] -> 2 h2o[c] + 2 co2[c] + 4 nadp[c] + 2 coa[c] + chcoa[c]	0
R22	1.1.1.100	B-ketoacyl-ACP synthase (c10,0), fatty acyl CoA synthase	6 h[m] + 4 nadph[m] + 3 malcoa[m] -> 2 h2o[m] + 2 co2[m] + 4 nadp[m] + 2 coa[m] + chcoa[m]	0

R23	1.5.1.3	Dihydrofolate reductase	h[c] + nadph[c] + dhf[c] -> thf[c] + nadp[c]	0
R24	1.5.1.3	Dihydrofolate reductase	2 h[c] + 2 nadph[c] + fot[c] -> 2 nadp[c] + thf[c]	0
R25	1.14.13.-	2-octaprenyl-6-methoxyphenol hydroxylase	o2[m] + nadph[m] + hm[m] -> h2o[m] + nadp[m] + h[m] + hmb[m]	0
R26	1.14.13.-	ubiquinone biosynthesis monooxygenase Coq7	hmmb[m] + o2[m] + nadph[m] + h[m] -> hmhmb[m] + nadp[m] + h2o[m]	0
R27	2.5.1.21	Squalene synthase	2 fpp[c] + nadph[c] + 3 h[c] -> 2 ppi[c] + sql[c] + nadp[c]	0
R28	1.14.13.132	squalene monooxygenase	sql[c] + o2[c] + nadph[c] + h[c] -> s23e[c] + nadp[c] + h2o[c]	0
R29	1.14.13.90	zeaxanthin epoxidase	atxin[c] + nadph[c] + h[c] + o2[c] -> vlxin[c] + nadp[c] + h2o[c]	0
R30	1.14.13.90	zeaxanthin epoxidase	zextin[c] + nadph[c] + h[c] + o2[c] -> axtin[c] + nadp[c] + h2o[c]	0
R31	1.1.1.133	dTDP-4-dehydrorhamnose reductase	dtdp6m[c] + h[c] + nadph[c] -> nadp[c] + dtdpdm[c]	0
R32	1.1.1.157	3-hydroxybutyryl-CoA dehydrogenase	c4hcoa[c] + nadp[c] <=> aaccoa[c] + nadph[c] + h[c]	0
R33	1.14.-.-	Sterigmatocystin biosynthesis monooxygenase	o2[m] + h[m] + nadph[m] + avn[m] -> h2o[m] + nadp[m] + havn[m]	0
R34	1.14.-.-	Sterigmatocystin biosynthesis monooxygenase	o2[c] + h[c] + nadph[c] + avn[c] -> h2o[c] + nadp[c] + havn[c]	0
R35	1.14.13.7	phenol 2-monooxygenase	3csol[m] + o2[m] + nadph[m] + h[m] -> 23dlne[m] + nadp[m] + h2o[m]	0
R36	1.14.13.7	phenol 2-monooxygenase	pnol[m] + o2[m] + nadph[m] + h[m] -> ccl[m] + nadp[m] + h2o[m]	0
R37	1.14.13.7	phenol 2-monooxygenase	rsnol[m] + o2[m] + nadph[m] + h[m] -> btol[m] + nadp[m] + h2o[m]	0
R38	1.14.13.7	phenol 2-monooxygenase	3csol[c] + o2[c] + nadph[c] + h[c] -> 23dlne[c] + nadp[c] + h2o[c]	0
R39	1.14.13.7	phenol 2-monooxygenase	pnol[c] + o2[c] + nadph[c] + h[c] -> ccl[c] + nadp[c] + h2o[c]	0
R40	1.14.13.7	phenol 2-monooxygenase	rsnol[c] + o2[c] + nadph[c] + h[c] -> btol[c] + nadp[c] + h2o[c]	0
R41	1.14.13.-	Oxidoreductases	4eol[m] + nadph[m] + h[m] + o2[m] -> 1eal[m] + nadp[m] + h2o[m]	0
R42	1.14.13.-	Oxidoreductases	bendl[m] + nadph[m] + h[m] + o2[m] -> btol[m] + nadp[m] + h2o[m]	0
R43	1.14.13.-	Oxidoreductases	sali[m] + nadph[m] + o2[m] + h[m] -> 25dhba[m] + nadp[m] + h2o[m]	0
R44	1.14.13.8	dimethylaniline monooxygenase	tafen[c] + o2[c] + nadph[c] + h[c] -> tafnox[c] + nadp[c] + h2o[c]	0

R45	1.14.13.8	dimethylaniline monooxygenase	tafen[p] + o2[p] + nadph[p] + h[p] -> tafnox[p] + nadp[p] + h2o[p]	0
R46	1.1.-.-	Aflatoxins biosynthesis;1,3,6,8-tetra hydroxy naphthalene reductase	h[c] + nadph[c] + vera[c] -> nadp[c] + dmst[c]	0
R47	1.1.-.-	Aflatoxins biosynthesis;1,3,6,8-tetra hydroxy naphthalene reductase	h[c] + nadph[c] + verb[c] -> h2o[c] + nadp[c] + dhdmst[c]	0
R48	1.1.1.100	beta-ketoacyl-[acyl-carrier protein](ACP) reductase	c4hacp[m] + nadp[m] <=> aacacp[m] + nadph[m] + h[m]	0
R49	1.1.1.100	beta-ketoacyl-[acyl-carrier protein](ACP) reductase	c6hacp[m] + nadp[m] <=> c6oacp[m] + nadph[m] + h[m]	0
R50	1.1.1.100	beta-ketoacyl-[acyl-carrier protein](ACP) reductase	c8hacp[m] + nadp[m] <=> c8oacp[m] + nadph[m] + h[m]	0
R51	1.1.1.100	beta-ketoacyl-[acyl-carrier protein](ACP) reductase	c10hacp[m] + nadp[m] <=> c10oacp[m] + nadph[m] + h[m]	0
R52	1.1.1.100	beta-ketoacyl-[acyl-carrier protein](ACP) reductase	c12hacp[m] + nadp[m] <=> c12oacp[m] + nadph[m] + h[m]	0
R53	1.1.1.100	beta-ketoacyl-[acyl-carrier protein](ACP) reductase	c14hacp[m] + nadp[m] <=> c14oacp[m] + nadph[m] + h[m]	0
R54	1.1.1.100	beta-ketoacyl-[acyl-carrier protein](ACP) reductase	c16hacp[m] + nadp[m] <=> c16oacp[m] + nadph[m] + h[m]	0
R55	1.1.1.100	3-oxoacyl-[acyl-carrier-protein] reductase	aacacp[c] + nadph[c] + h[c] <=> c4hacp[c] + nadp[c]	0.162
R56	2.3.1.86	fatty-acyl-CoA synthase	c4dacp[c] + nadph[c] + h[c] <=> c40acp[c] + nadp[c]	0.162
R57	1.1.1.100	3-oxoacyl-[acyl-carrier-protein] reductase	c6oacp[c] + nadph[c] + h[c] <=> c6hacp[c] + nadp[c]	0.162
R58	2.3.1.86	fatty-acyl-CoA synthase	c6dacp[c] + nadph[c] + h[c] <=> c60acp[c] + nadp[c]	0.162
R59	1.1.1.100	3-oxoacyl-[acyl-carrier-protein] reductase	c8oacp[c] + nadph[c] + h[c] <=> c8hacp[c] + nadp[c]	0.162
R60	2.3.1.86	fatty-acyl-CoA synthase	c8dacp[c] + nadph[c] + h[c] <=> c80acp[c] + nadp[c]	0.162
R61	1.1.1.100	3-oxoacyl-[acyl-carrier-protein] reductase	c10oacp[c] + nadph[c] + h[c] <=> c10hacp[c] + nadp[c]	0.162
R62	2.3.1.86	fatty-acyl-CoA synthase	c10dacp[c] + nadph[c] + h[c] <=> c100acp[c] + nadp[c]	0.162
R63	1.1.1.100	3-oxoacyl-[acyl-carrier-protein] reductase	c12oacp[c] + nadph[c] + h[c] <=> c12hacp[c] + nadp[c]	0.162
R64	2.3.1.86	fatty-acyl-CoA synthase	c12dacp[c] + nadph[c] + h[c] <=> c120acp[c] + nadp[c]	0.162
R65	1.1.1.100	3-oxoacyl-[acyl-carrier-protein] reductase	c14oacp[c] + nadph[c] + h[c] <=> c14hacp[c] + nadp[c]	0.162
R66	2.3.1.86	fatty-acyl-CoA synthase	c14dacp[c] + nadph[c] + h[c] <=> c140acp[c] + nadp[c]	0.162
R67	1.1.1.100	3-oxoacyl-[acyl-carrier-protein] reductase	c16oacp[c] + nadph[c] + h[c] <=> c16hacp[c] + nadp[c]	0.162
R68	2.3.1.86	fatty-acyl-CoA synthase	c16dacp[c] + nadph[c] + h[c] <=> c160acp[c] + nadp[c]	0.162

R69	1.1.1.330	very-long-chain 3-oxoacyl-CoA reductase	c18ocoa[c] + nadph[c] + h[c] <=> c18hcoa[c] + nadp[c]	0.156
R70	1.3.1.93	very-long-chain enoyl-CoA reductase	c18dcoa[c] + nadph[c] + h[c] <=> c180coa[c] + nadp[c]	0.156
R71	1.1.1.330	very-long-chain 3-oxoacyl-CoA reductase	c20ocoa[c] + nadph[c] + h[c] <=> c20hcoa[c] + nadp[c]	0.002
R72	1.3.1.93	very-long-chain enoyl-CoA reductase	c20dcoa[c] + nadph[c] + h[c] <=> c200coa[c] + nadp[c]	0.002
R73	1.1.1.330	very-long-chain 3-oxoacyl-CoA reductase	c22ocoa[c] + nadph[c] + h[c] <=> c22hcoa[c] + nadp[c]	0.001
R74	1.3.1.93	very-long-chain enoyl-CoA reductase	c22dcoa[c] + nadph[c] + h[c] <=> c220coa[c] + nadp[c]	0.001
R75	1.1.1.330	very-long-chain 3-oxoacyl-CoA reductase	c24ocoa[c] + nadph[c] + h[c] <=> c24hcoa[c] + nadp[c]	0
R76	1.3.1.93	very-long-chain enoyl-CoA reductase	c24dcoa[c] + nadph[c] + h[c] <=> c240coa[c] + nadp[c]	0
R77	1.14.13.70	sterol 14-demethylase	lnst[c] + 3 o2[c] + 3 nadph[c] + 2 h[c] -> dctol[c] + for[c] + 3 nadp[c] + 4 h2o[c]	0
R78	1.3.1.72	lanosterol delta24-reductase	lnst[c] + nadph[c] + h[c] -> dhstro[c] + nadp[c]	0
R79	1.3.1.70	delta14-sterol reductase;C-14 sterol reductase (cG24)	h[c] + nadph[c] + dctol[c] -> nadp[c] + dcdol[c]	0
R80	1.14.13.72	methylsterol monooxygenase;C-4 sterol methyl oxidase (cG25)	3 o2[c] + 2 h[c] + 3 nadph[c] + dcdol[c] -> 4 h2o[c] + 3 nadp[c] + dcda[c]	0
R81	1.1.1.270	3-keto-steroid reductase	h[c] + nadph[c] + cdol[c] -> nadp[c] + mzymst[c]	0
R82	1.3.1.72	delta24-sterol reductase	zymst[c] + nadph[c] + h[c] -> ac8bol[c] + nadp[c]	0
R83	1.14.21.6	lathosterol oxidase	ac7bol[c] + nadph[c] + h[c] + o2[c] -> pvd3[c] + nadp[c] + 2 h2o[c]	0
R84	1.3.1.72	delta24-sterol reductase	acdbol[c] + nadph[c] + h[c] -> ac7bol[c] + nadp	0
R85	1.3.1.21	7-dehydrocholesterol reductase	pvd3[c] + nadph[c] + h[c] -> chtrol[c] + nadp[c]	0
R86	1.3.1.72	delta24-sterol reductase	7strol[c] + nadph[c] + h[c] -> pvd3[c] + nadp[c]	0
R87	1.14.13.70	sterol 14-demethylase	obl[c] + 3 o2[c] + 3 nadph[c] + 2 h[c] -> amt3bol[c] + for[c] + 3 nadp[c] + 4 h2o[c]	0
R88	1.3.1.70	Delta14-sterol reductase	amt3bol[c] + nadph[c] + h[c] -> mefol[c] + nadp[c]	0
R89	1.3.1.21	7-dehydrocholesterol reductase	dhrsol[c] + nadph[c] + h[c] -> isol[c] + nadp[c]	0
R90	1.3.1.71	delta24(24(1))-sterol reductase	h[c] + nadph[c] + egteol[c] -> nadp[c] + egstr[c]	0
R91	1.14.21.6	C-5 sterol desaturase (ERG3)	nadph[c] + epst[c] -> 3 h[c] + nadp[c] + ergod[c]	0
R92	1.14.21.6	lathosterol oxidase	acdbol[c] + nadph[c] -> 7dstrol[c] + nadp[c] + 3 h[c]	0
R93	1.3.1.21	7-dehydrocholesterol reductase	7dstrol[c] + nadph[c] + h[c] -> nadp[c] + dmstrol[c]	0
R94	1.1.1.101	Acylglycerone-phosphate reductase	4 h[c] + nadph[c] + at3p2[c] -> nadp[c] + agl3p[c]	0
R95	1.1.1.102	3-Dehydrosphinganine reductase	h[c] + nadph[c] + dhsph[c] -> nadp[c] + sph[c]	0
R96	1.14.-.-	C4-hydroxylase	sph[c] + nadph[c] + h[c] + o2[c] -> nadp[c] + psph[c] + h2o[c]	0

R97	1.14.-.-	C4-hydroxylase	o2[c] + h[c] + nadph[c] + dcer2[c] -> h2o[c] + nadp[c] + pcer2[c]	0
R98	1.3.1.27	hexadecanal:NADP+ delta2-oxidoreductase	c16e[c] + nadph[c] + h[c] -> c16a[c] + nadp[c]	0
R99	1.14.-.-	sphingolipid delta-4 desaturase	o2[c] + h[c] + nadph[c] + dcer2[c] -> h2o[c] + nadp[c] + pcer2[c]	0
R100	1.14.14.1	dimethylallyl diphosphate	c204(6)[c] + o2 + nadph[c] + 2 h[c] -> 1415eet[c] + nadp[c] + h2o[c]	0
R101	1.14.14.1	Cytochrome P450	c204(6)[c] + o2 + nadph[c] + 2 h[c] -> 1112eet[c] + nadp[c] + h2o[c]	0
R102	1.14.14.1	Cytochrome P450	c204(6)[c] + o2 + nadph[c] + 2 h[c] -> 89eet[c] + nadp[c] + h2o[c]	0
R103	1.14.14.1	Cytochrome P450	c204(6)[c] + o2 + nadph[c] + 2 h[c] -> 56eet[c] + nadp[c] + h2o[c]	0
R104	1.14.14.1	Cytochrome P450	c204(6)[c] + o2 + nadph[c] + 2 h[c] -> 16hete[c] + nadp[c] + h2o[c]	0
R105	1.14.19.1	delta 9 desaturase	c180acp[m] + o2[m] + nadph[m] + 3 h[m] -> c181acp[m] + 2 h2o[m] + nadp[m]	0
R106	1.6.2.4	NADPH--cytochrome P450 reductase	nadph[c] + 2 feri[m] -> h[m] + nadp[c] + 2 fero[m]	0
R107	1.14.-	C4-hydroxylase	sph[c] + nadph[c] + h[c] + o2[c] -> nadp[c] + psph[c] + h2o[c]	0
R108	1.14.19.1	delta 9 desaturase	c160coa[c] + o2[c] + nadph[c] + h[c] -> c161coa[c] + 2 h2o[c] + nadp[c]	0
R109	1.14.19.1	delta 9 desaturase	c180coa[c] + o2[c] + nadph[c] + h[c] -> c181coa[c] + 2 h2o[c] + nadp[c]	0.152
R110	1.14.19.-	delta 12 desaturase	c181coa[c] + o2[c] + nadph[c] + h[c] -> c182coa[c] + 2 h2o[c] + nadp[c]	0.142
R111	1.14.19.-	delta 15 desaturase	c182coa[c] + o2[c] + nadph[c] + h[c] -> c183(3)coa[c] + 2 h2o[c] + nadp[c]	0
R112	1.14.19.3	delta 6 desaturase	c182coa[c] + o2[c] + nadph[c] + h[c] -> c183(6)coa[c] + nadp[c] + 2 h2o[c]	0.139
R113	1.14.19.3	delta 6 desaturase	c183(3)coa[c] + o2[c] + nadph[c] + h[c] -> c184(3)coa[c] + nadp[c] + 2 h2o[c]	0
R114	1.14.19.1	delta 9 desaturase	c200coa[c] + o2[c] + nadph[c] + h[c] -> c201coa[c] + 2 h2o[c] + nadp[c]	0
R115	1.1.1.330	very-long-chain 3-oxoacyl-CoA reductase	c202ocoa[c] + nadph[c] + h[c] <=> c202hcoa[c] + nadp[c]	0

R116	1.3.1.93	very-long-chain enoyl-CoA reductase	c202dcoa[c] + nadph[c] + h[c] <=> c202coa[c] + nadp[c]	0
**R117**	**1.1.1.330**	**very-long-chain 3-oxoacyl-CoA reductase**	**c203(6)ocoa[c] + nadph[c] + h[c] <=> c203(6)hcoa[c] + nadp[c]**	**0.135**
**R118**	**1.3.1.93**	**very-long-chain enoyl-CoA reductase**	**c203(6)dcoa[c] + nadph[c] + h[c] <=> c203(6)coa[c] + nadp[c]**	**0.135**
R119	1.1.1.330	very-long-chain 3-oxoacyl-CoA reductase	c204(3)ocoa[c] + nadph[c] + h[c] <=> c204(3)hcoa[c] + nadp[c]	0
R120	1.3.1.93	very-long-chain enoyl-CoA reductase	c204(3)hcoa[c] <=> c204(3)dcoa[c] + h2o[c]	0
**R121**	**1.14.19.-**	**delta 5 desaturase**	**c203(6)coa[c] + o2[c] + nadph[c] + h[c] -> c204(6)coa[c] + nadp[c] + 2 h2o[c]**	**0.135**
R122	1.14.19.-	delta 5 desaturase	c204(3)coa[c] + 2 o2[c] + nadph[c] + 3 h[c] -> c205(3)coa[c] + nadp[c] + 4 h2o[c]	0
R123	1.14.19.-	delta 15 desaturase	c204(6)coa[c] + o2[c] + nadph[c] + h[c] -> c205(3)coa[c] + 2 h2o[c] + nadp[c]	0
R124	1.1.1.330	very-long-chain 3-oxoacyl-CoA reductase	c225(3)ocoa[c] + nadph[c] + h[c] <=> c225(3)hcoa[c] + nadp[c]	0
R125	1.3.1.93	very-long-chain enoyl-CoA reductase	c225(3)hcoa[c] <=> c225(3)dcoa[c] + h2o[c]	0
R126	1.1.1.330	very-long-chain 3-oxoacyl-CoA reductase	c245(3)ocoa[c] + nadph[c] + h[c] <=> c245(3)hcoa[c] + nadp[c]	0
R127	1.3.1.93	very-long-chain enoyl-CoA reductase	c245(3)hcoa[c] <=> c245(3)dcoa[c] + h2o[c]	0
R128	1.14.19.3	delta 6 desaturase	c245(3)coa[c] + o2[c] + nadph[c] + h[c] -> c246(3)coa[c] + nadp[c] + 2 h2o[c]	0
**R129**	**1.2.1.38**	**N-Acetyl-gamma-glutamyl-phosphate reductase**	**2 h[m] + nadph[m] + naglup[m] <=> nadp[m] + naglus[m] + pi[m]**	**0.552**
R130	1.2.1.11	aspartate-semialdehyde dehydrogenase	2 h[c] + nadph[c] + basp[c] <=> nadp[c] + pi[c] + aspsa[c]	0
R131	1.1.1.3	homoserine dehydrogenase	h[m] + nadph[m] + aspsa[m] <=> nadp[m] + hser[m]	0
R132	1.1.1.3	homoserine dehydrogenase	h[c] + nadph[c] + aspsa[c] <=> nadp[c] + hser[c]	0
R133	1.2.1.18	malonate-semialdehyde dehydrogenase	oppa[m] + nadp[m] + coa[m] <=> h[m] + nadph[m] + malcoa[m]	0
R135	1.5.1.20	methylenetetrahydrofolate reductase [NAD(P)H]	h[c] + nadph[c] + metthf[c] <=> nadp[c] + mthf[c]	0
**R136**	**1.1.1.34**	**hydroxymethylglutaryl-CoA reductase (NADPH)**	**2 h[c] + 2 nadph[c] + hmgcoa[c] <=> 2 nadp[c] + coa[c] + mvl[c]**	**0.162**
